# Comparative Genomic and Phylogenetic Analysis of the First Usutu Virus Isolate from a Human Patient Presenting with Neurological Symptoms

**DOI:** 10.1371/journal.pone.0064761

**Published:** 2013-05-31

**Authors:** Paolo Gaibani, Francesca Cavrini, Ernest A. Gould, Giada Rossini, Anna Pierro, Maria Paola Landini, Vittorio Sambri

**Affiliations:** 1 Operative Unit of Clinical Microbiology, Regional Reference Centre for Microbiological Emergencies (CRREM), St.Orsola-Malpighi University Hospital, Bologna, Italy; 2 UMR190 Emergence des Pathologies Virales, Unité des Virus Emergents, Faculté de Médecine de Marseille, Marseille, France; 3 Unit of Microbiology, Central Laboratory “Area Vasta Romagna”, Pievesestina, Cesena, Italy; Columbia University, United States of America

## Abstract

Usutu virus (USUV) is a mosquito-borne flavivirus, belonging to the Japanese encephalitis antigenic complex, that circulates among mosquitoes and birds. We describe and analyze the complete genome sequence of the first USUV strain isolated from an immunocompromised patient with neuroinvasive disease. This USUV isolate showed an overall nucleotide identity of 99% and 96%, respectively, with the genomes of isolates from Europe and Africa. Comparison of the human USUV complete polyprotein sequence with bird-derived strains, showed two unique amino acid substitutions. In particular, one substitution (S595G) was situated in the DIII domain of the viral Envelope protein that is recognized by flavivirus neutralizing antibodies. An additional amino acid substitution (D3425E) was identified in the RNA-dependent RNA polymerase (RdRp) domain of the NS5 protein. This substitution is remarkable since E3425 is highly conserved among the other USUV isolates that were not associated with human infection. However, a similar substitution was observed in Japanese encephalitis and in West Nile viruses isolated from humans. Phylogenetic analysis of the human USUV strain revealed a close relationship with an Italian strain isolated in 2009. Analysis of synonymous nucleotide substitutions (SNSs) among the different USUV genomes showed a specific evolutionary divergence among different countries. In addition, 15 SNSs were identified as unique in the human isolate. We also identified four specific nucleotide substitutions in the 5′ and 3′ untranslated regions (UTRs) in the human isolate that were not present in the other USUV sequences. Our analyses provide the basis for further experimental studies aimed at defining the effective role of these mutations in the USUV genome, their potential role in the development of viral variants pathogenic for humans and their evolution and dispersal out of Africa.

## Introduction

Usutu virus (USUV), an arthropod-borne flavivirus within the Japanese encephalitis virus antigenic complex, is closely related to several human and animal pathogenic members of this complex including West Nile virus (WNV), Murray Valley Encephalitis virus (MVEV) and Japanese encephalitis virus (JEV) [1]. USUV is maintained in a typical enzootic cycle between mosquitoes and birds [2]. It was originally identified in South African mosquito species in 1959 but during the past >17 years, the virus has apparently dispersed out of Africa into Europe [3]–[5], presumably carried by migratory birds and their associated mosquitoes [6]–[12]. In particular, a recent study reported the first evidence of USUV introduction in Italy in 1996 [5]. Ten years later, an elevated rate of USUV circulation was reported in vectors and birds in Italy [13]–[16]. Following its dispersal in parts of western and central Europe, many studies have investigated the epidemiology and pathogenicity of USUV for wild birds which appear to be the predominant vertebrate host species. USUV infection of birds is often characterized by encephalitis, myocardial degeneration, and necrosis of the liver and spleen [6]–[9]. USUV is not considered to be a significant human pathogen [10], [17]–[18]. The first reported cases of human USUV infection were observed in Africa in two patients presenting with fever, rash and jaundice [3]. Subsequently, two USUV-positive cases of meningoencephalits were reported in immunecompromised patients in Italy [17]–[19]. Recently, USUV RNA was detected using real-time RT-PCR in cerebrospinal fluid specimens of three immunocompetent patients suffering from meningoencephalits [20]. Even more recently, the first 5 cases of USUV specific-IgG positive sera in healthy blood donors, were reported confirming that USUV actively circulates amongst asymptomatic subjects in Europe [21]–[22].

Similar to other flaviviruses, USUV is a spherical, small enveloped virus with a diameter 40–60 nm. The genome is a single-stranded positive sense RNA of 11 Kb, with a 5′ cap structure [7], that contains one large open reading frame (ORF) comprising nucleotides (nt) 97 to 10401, encoding a unique polyprotein of 3434 amino acids (aa). The USUV polyprotein is processed post-translationally by cellular and viral proteases into three structural proteins: capsid (97–474), pre-membrane/membrane (475–975) and envelope (976–2475) and seven non-structural proteins, NS1 (2476–3531), NS2a (3532–4212), NS2b (4213–4605), NS3 (4604–6462), NS4a (6463–6840), NS4b (6910–7684) and NS5 (7684–10398) [7].

Previous molecular studies on USUV isolates from vectors and birds in Hungary (Budapest), Austria (Vienna and Meise H), Germany (Mannheim), and South Africa (SAAR-1776) revealed significant differences, both in the nt and aa sequences [7], [11]–[13].

The aim of this study was to sequence the complete genome of the first human-encephalitic USUV isolate [17] and to analyse it comparatively with these non-human strains of USUV to see whether or not significant differences could be identified.

Conventionally, synonymous nucleotide substitutions (SNS), also called silent substitutions, are considered as selectively neutral or subject to minor constraints among the virus variants [23]. However, during the recent past, several studies have demonstrated that nt substitutions in the 5′ and 3′ untranslated regions (UTRs) may have an impact on viral replication and infectivity by altering the secondary structure of the viral RNA [24]–[26]. Studies have also focused on the effective role of synonymous nucleotide substitutions within the coding region of the viral RNA, demonstrating a notable impact on the viral phenotype by altering RNA folding or codon usage [22], [26]–[29]. Current evidence suggests that the impact of these SNS is more apparent in viruses with compact genomes such as the ssRNA viruses, including the flaviviruses [23], [31]. Based on these interpretations, we have investigated the presence of SNS among the different USUV isolates in the hope of understanding their likely role in the evolution of USUV and its pathogenicity.

## Materials and Methods

### Virus Isolation

Usutu virus was isolated from the plasma of a patient who developed neuroinvasive disease following an orthotropic liver transplantation (OLT) [17]. The plasma was positive when tested by WNV transcription-mediated amplification assay (TMA) [19]. The TMA positive plasma was inoculated (100 µl) onto confluent cell culture monolayers (African green monkey kidney, Vero E6) [21] in a 24-well cell culture plate and centrifuged at 3,000 rpm for 12 hours. The infected cells were then washed to remove residual RNA, incubated at 37°C in the presence of 5% CO_2_ and observed daily until cytopathic effects (CPE) were detected on day 14 post-infection. Confirmation of the presence of USUV RNA was established using real-time RT-PCR, as previously described [20].

### Genome Sequence and Analysis of the USUV Human Isolate

The viral RNA was extracted from 200 µl of supernatant medium from Vero E6 infected cells using an automated nucleic acids extractor NucliSens EasyMag (Biomerieux, France) according to the manufacture’s protocol. Degenerate primer pairs [7] were used to produce overlapping RT-PCR products covering the entire genome of USUV. RT-PCR assays were performed using the Superscript One Step RT-PCR with Platinum Taq Kit (Invitrogen, UK). Each 50 µl reaction mixture contained 25 µl of 2X Reaction Mix (containing 0,4 mM of each dNTP and 2,4 mM MgSO_4_), 25 pmol of the appropriate forward and reverse primers, 1 of enzyme mix and 5 µl of template RNA. The amplification conditions were: 30 min at 55°C, 2 min at 94°C, 40 cycles of 15s at 94°C, 30s at 57°C, 1 min at 72°C and finally 7 min at 72°C. For each RT-PCR assay the agarose gel electrophoresis analysis revealed a single band of expected size. For each PCR product obtained, both strands of the amplicon were sequenced using RT-PCR and sequencing primers, previously described [7]. For each sequencing reaction the electropherogram was analysed by Chromas lite software V2.01 package (available at www.technelysium.com.au) and the nucleotide sequences were aligned and confirmed using BLAST software (http://www.ncbi.nlm.nih.gov/blast). The human USUV complete genome sequence (designated Bologna/09) was deposited in GenBank with the following accession number: **HM569263**. Multiple alignment of the USUV complete genome sequences derived from the published isolates, was generated with ClustalW software (version 2, available at: http://www.ebi.ac.uk/clustalw2). To evaluate the presence of single nucleotide polymorphisms, the human USUV genome was compared with genomic sequences of the following USUV isolates derived from birds and vectors: Austrian (Vienna 2001 [**AY453411**] and Meise H [**JQ219843**]), Hungarian (Budapest 2005 [**EF206350**]), Germany (Mannheim [**HE599647**]), Italian 2009 ([**JF266698**]), African (South Africa-1776 [**AY453412**]). Moreover, the genome of the Bologna/09 strain was compared with genomic sequences of flavivirus strains: WNV lineage 1 **[JN858069]**, WNV lineage 2 **[JN858070]**, JEV **[NC001437]**, DENV1 **[AF309641]**, DENV2 **[FJ744705]**, DENV3 **[FJ74309641]**, DENV4 **[MI4931]**, YFV **[NC002031]**, MVEV **[MC000943]**.

### Phylogenetic Analysis

Phylogenetic analysis was performed by aligning the complete nucleotide sequences of the poly-protein coding regions of the Bologna/09 strain with the complete coding region of the USUV isolates available in GenBank (**AY453411, JQ219843, EF206350, HE599647, JF266698, AY453412)**. Phylogenetic trees were inferred using nucleotide alignment [32]. Neighbor joining (NJ) method [30] was performed and implemented in MEGA software (v 4.1) [33]. The most parsimonious tree incorporated 1000 bootstrap replications.

### Analysis of 5′ and 3′ Untranslated Regions (UTR)

For the analysis of the 5′ and 3′ UTR, the partial or complete sequences of the two distal regions from all the USUV genomes present in GenBank (Vienna, Meise H, Budapest, Manheim, Italian, South African and Bologna/09) were aligned using ClustalW software and corrected manually. To identify the presence of single nucleotide mutations between the different USUV isolates and the human isolate, the nucleotide sequence of the first 96 and the last 666 nucleotides of the different genomes, corresponding respectively to the 5′ and 3′ UTR, were used for the analysis. To compare the stability of the deduced secondary structure of the 5′ and 3′ terminal sequences among the different strains, the free energy (Kcal/ml) of each isolate was calculated by CLC program (CLC RNA Workbench, v4.1, Denmark).

## Results

### Genome Analysis

**Figure 1:**
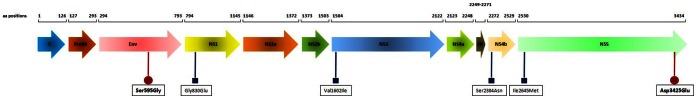
ORF of the USUV genome. Single genes and relative amino acid sizes are shown along each region on the USUV genome. C, capsid gene; PreM, PreMembrane gene; Env, envelope gene; NS, no-structural genes. Comparison of amino-acid substitutions between human isolate (Bologna/09) and African (SAAR-1776 strain) and European (Vienna 2001, Meise H 2002, Budapest 2005, Italia 2009 and Germany 2011) USUV isolates. Blue square corresponding to the aa mutations unique for the Italian isolates (Bologna/09 and Italy 2009). Red circle showing the aa mutations of human isolate (Bologna/09).

**Figure 2:**
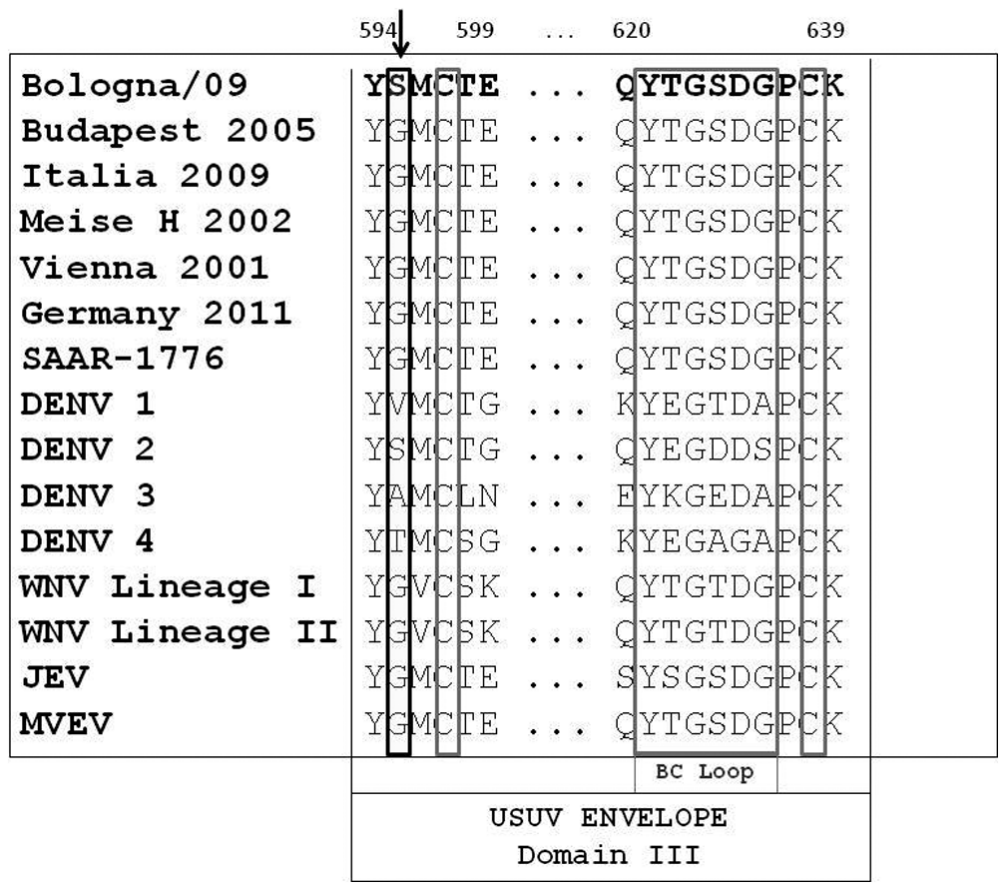
Amino acid sequence alignment of the flavivirus domain III in the envelope region, corresponding to the residues 302–337. The conserved cysteine and BC loop are boxed with grey lines. The arrow indicates the S595G mutation of Bologna/09 and their corresponding amino acid in the USUV isolates and in the JEV viruses complex, boxed with a black line.

We determined the full-length nucleotide sequence of Bologna/09, andconducted an extensive comparative analysis of the human isolate with other USUV strains. Comparison of the complete genome nucleotide sequence with that of selected closely related mosquito-borne viruses, including WNV Lineage I and II, JEV, DENV 1-4, YFV, MVEV, revealed a percentage homology of less than 73% as shown in Table 1. The Usutu viruses isolated in Europe displayed 98% to 100% identity within the different strains, including the Bologna/09 isolate among the different nucleotide coding regions (Table 1). In contrast, the SAAR strain, isolated from South Africa showed lower nucleotide identity (between 95% and 98%) in the different coding regions. To evaluate the amino acid substitutions common to Bologna/09 and the other USUV isolates, the amino acid variation was assessed (Figure 1). A description of the triplet codons and deduced amino acid differences is shown in the supplementary Table S1. Interestingly, comparison of the amino acid sequences of Bologna/09 with the non-human USUV strains revealed two unique substitutions, one in the envelope and the other in the NS5 gene (Figure 1). To visualize the single amino acid substitutions among the different USUV genomes, a profile alignment with the different amino acid changes is shown in Table S1. In particular, the Bologna/09 strain showed a unique single amino acid substitution (S595G) in the DIII-Ir domain when compared with all other USUV isolates (as shown in Figure 2) and with the other flaviviruses belonging to the JEV serogroup. Interestingly, a similar amino acid was identified in several DENV-2 strains (Figure 2). The DIII-lr domain is highly variable among different flaviviruses [34]. Previous studies indicated that neutralizing, virus-type-specific antibodies recognize the DIII-Ir domain [35]–[36].

**Table 1:** Nucleotide identities of entire polyprotein coding region (structural and not structural protein) of Bologna/09 isolate in comparison to the different USUV strains and the most representative JEV serogroup complex viruses.

	Nucleotide identity (%) of USUV Bologna/09													
Strains														Entire ORF(%)
	C	pr/M	E	NS1	NS2a	NS2b	NS3	NS4a	2K	NS4b	NS5	5′-UTR	3′-UTR	
**USUV Strains**														
**SAAR-1776** **(AY453412)**	98	97	95	96	97	96	97	96	97	96	97	96	96	**96**
**Vienna 2001** **(AY453411)**	99	98	99	99	99	99	99	100	99	99	99	98	96	**99**
**Budapest 2005** **(EF206350)**	99	98	99	99	99	100	99	99	98	99	99	98	99	**99**
**Italia 2009** **(JF266698)**	99	99	99	99	99	100	99	99	98	99	99	98	99	**99**
**Flavivirus Strains**														
**WNV Lineage 1** **(JN858069)**	63	68	69	68	64	67	71	70	76	63	73	64	64	**69**
**WNV Lineage 2** **(JN858070)**	63	68	69	68	64	66	71	69	71	62	72	61	50	**69**
**JEV** **(NC001437)**	73	69	70	46	69	71	74	71	78	69	74	62	75	**72**
**DENV1** **(AF309641)**	53	48	55	56	42	46	60	51	50	49	64	41	58	**56**
**DENV2** **(FJ744705)**	54	48	52	44	43	51	61	49	46	46	63	47	56	**56**
**DENV3** **(FJ74309641)**	55	47	55	43	42	47	59	51	46	51	64	60	55	**56**
**DENV4** **(M14931)**	54	50	55	41	45	45	61	52	49	48	63	51	60	**56**
**YFV** **(NC002031)**	42	48	54	42	46	45	55	52	55	44	61	43	45	**53**
**MVEV** **(MC000943)**	73	75	73	71	68	72	74	72	76	67	74	66	76	**73**

In addition, the amino acid sequence of the NS5 protein obtained from Bologna/09 strain revealed a single amino acid substitution (D3425E) not present in any of the non-human USUV isolates (Figure 1), although a similar amino acid substitution was identified in all JEV, WNV (Lineage 1 and 2), MVEV and Kunjin strains that were analysed. Comparative analysis of the complete coding region of all the USUV strains revealed four amino acid substitutions common to all Italian isolates at positions G830E, V1602I, S2304N and I2645M. However, the human Bologna/09 isolate also had the two additional unique substitutions referred to above. These six amino acid substitutions are defined in Figure 1.

### Phylogenetic Analysis Based on the Complete Coding Region Sequences of the USUV Genome

**Figure 3:**
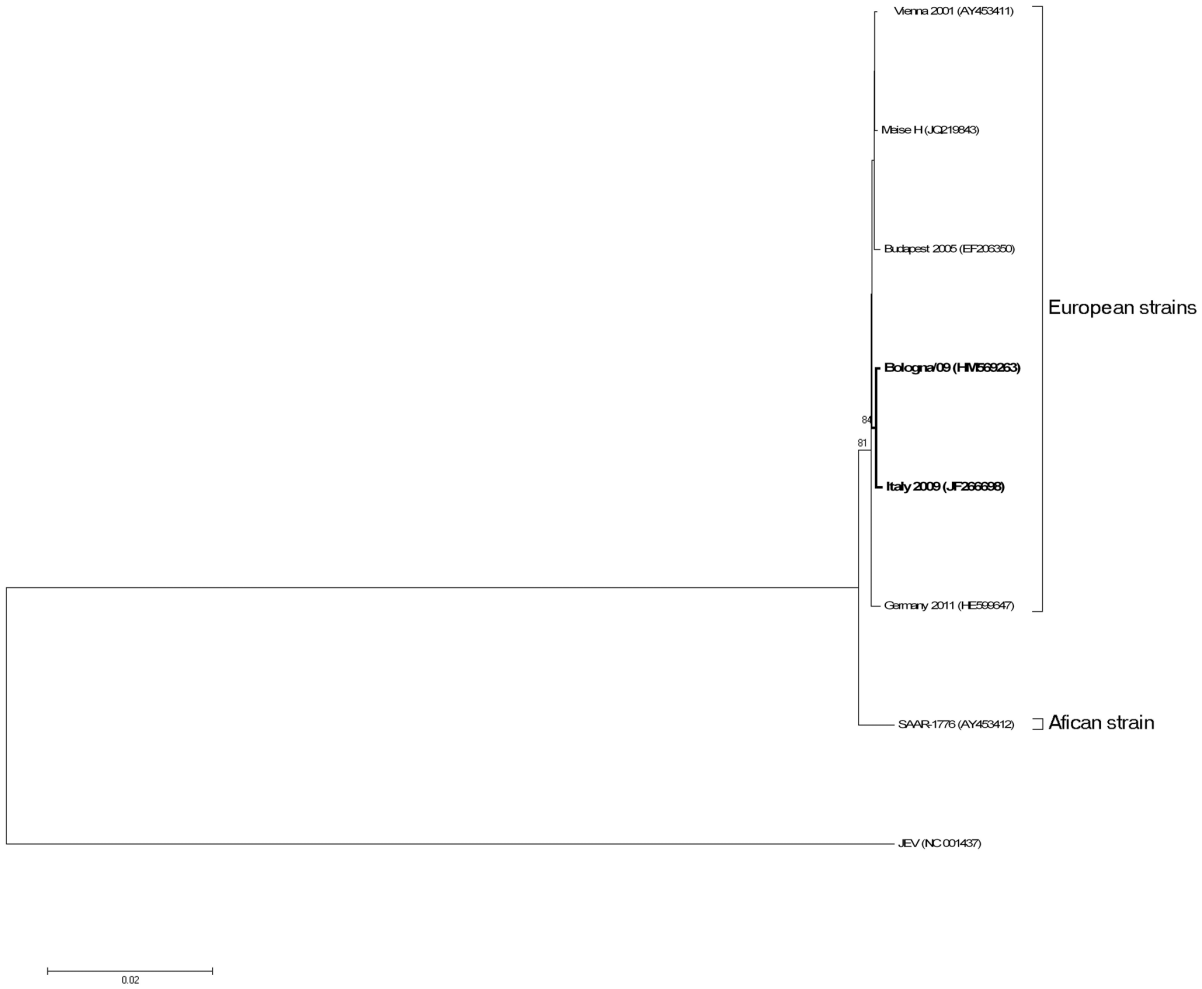
Phylogenetic analysis of USUV strain isolated from human plasma (Bologna/09) and other USUV isolates. The Phylogenetic tree inferred using the Neighbor-Joining method based on the complete nucleotide polyprotein gene coding region of different USUV strains. The evolutionary distances were computed using the Maximum Composite Likelihood method and are in the units of the number of base substitutions per site. The tree was rooted by using SAAR-1776 strain isolated in South Africa, as the outgroup virus. Significant bootstrap values (>80%) are indicated.

A phylogenetic tree of the entire nucleotide coding region was constructed in order to investigate and compare the evolutionary relationships between Bologna/09 and the other USUV isolates. (Figure 3). The tree showed close relationships among the European isolates and confirmed the contrasting nucleotide and amino acid divergence of the SAAR isolate (Figure 3). A strain of JEV was used as the out-group.

### Analysis of Synonymous Nucleotide Substitutions

**Figure 4:**
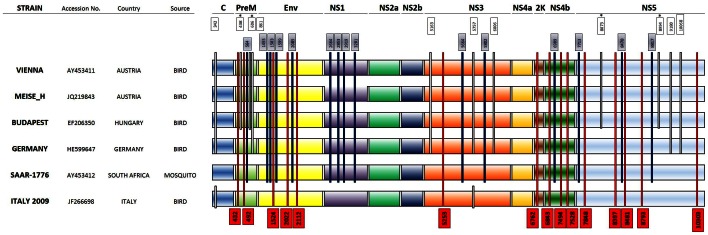
Synonymous nucleotide substitutions (SNS) over the entire coding region of the seven USUV strains. Black bars indicate the SNS unique for the human isolate (Bologna/09), while the grey bars indicate the SNS unique for the Italian isolates (Bologna/09 and Italy 2009). The asterisks indicate the unique SNS for the Austrian-Hungarian strains (Budapest 2005, Vienna 2001, Meise_H). The corresponding nucleotide position over the entire coding region is indicated above each bar.

**Figure 5:**
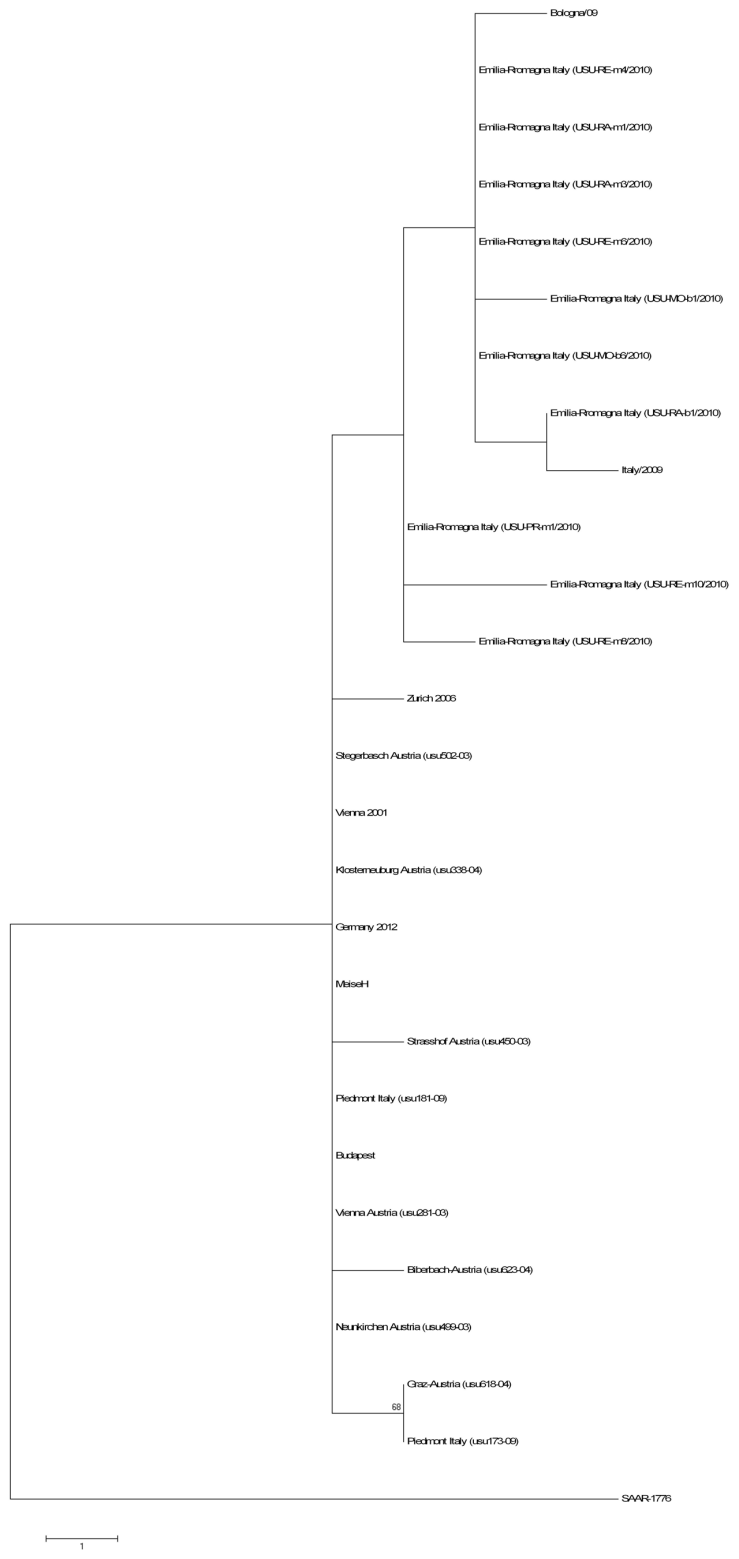
Maximum Parsimony phylogenetic analysis of polyprotein Env gene fragment sequences calculated for the Austrian isolates: Meise_H (JQ219843), Vienna 2001 (AY453411), usu502-03 (EF078297), usu281-03 (EF078294), usu623-04 (EF078300), usu618-04 (EF078299), usu499-03 (EF078296), usu338-04 (EF078298), usu450-03 (EF078295). **Italian isolates from Piedmont region (West part of Italy): usu090-10 (JN257974), usu181-09 (JN257976), usu173-09 (JN257975).** Italian isolates from Emilia-Romagna region (East part of Italy): USU-MO-b1/2010 (JF834683), USU-MO-b6/2010 (JF834661), USU-RE-m4/2010 (JF834673), USU-RE-m6/2010 (JF834671), USU-RE-m8/2010 (JF834675), USU-RE-m10/2010 (JF834677), USU-RA-m3/2010 (JF834667), USU-RA-m1/2010 (JF834665), USU-RA-b1/2010 (JF834669), USU-PR-m1/2010 (JF834663), Bologna/09 (HM569263), Italy 2009 (JF266698). German: Germany 2011 (HE599647), Hungarian: Budapest 2005 (EF206350) and South-African: SAAR-1776 (AY453412) isolates. Bootstrap values >50% are displayed.

Comparative analysis of the ORF of the USUV isolates revealed 41 Synonymous Nucleotide Substitutions (SNS) (Figure 4). Interestingly these SNS identified two clusters of genetic variants amongst the USUV isolates, viz., Italian and Austrian-Hungarian clusters. In particular, the Italian group showed 15 unique SNS distributed throughout the entire coding region (G564A, G1455A, T1503C, G1599A, C2085T, G2664A, A2853C, T2919C, T3201C, T 6909C, G7713A, C8478T, C8817T). Importantly, all SNS in the NS1 gene were unique to the Italian isolates (Figure 4). In contrast, the Austrian-Hungarian group showed only 4 unique SNS throughout the entire coding region (G438A, T606C, C8073T and T8994C). On the basis of these specific nucleotide substitutions, a phylogenetic analysis based on the *Env* region (between nucleotide positions 1180 to 1585) obtained from different USUV partial sequences available in GenBank was performed in order to confirm our previous results. The Maximum-likelihood algorithm confirmed the different clustering of Italian and Austrian-Hungarian groups (Figure 5). However, comparison of partial nucleotide sequences of the *Env* region obtained from the Italian strains isolated from mosquitoes and birds showed that the SNS at position G1599A was not present in all Italian strains. In detail, all strains isolated from the Piedmont region (West-Part of Italy) showed similar SNS to the Austrian-Hungarian strains, while in most of the Emilia-Romagna strains this specific SNS was not found. Based on these results, a Maximum-Parsimony phylogenetic analysis confirmed the clustering of the isolates collected from the West part of Italy to the Austrian-Hungarian strains, while the all the Emilia-Romagna isolates clustered with the Bologna/09 and the Italy 2009 isolates (East part of Italy) (Figure 5).

Furthermore, 27 SNS were identified in the human isolate (Bologna/09) distributed throughout the entire coding region, 15 of which were unique for this virus (C432T, T492C, G1524A, C2022T, C2112T, T5253G, T6762C, T6843A, A7494G, C7528T, G7848A, A8397G, T8481C, C8793T, C10303T). Most of these were found in the Non-Structural proteins (10 out of 15 unique SNS) and also in the 2K, NS4b and NS5 proteins (Figure 4).

### Analysis of 5′ and 3′ UTR Regions

**Figure 6:**
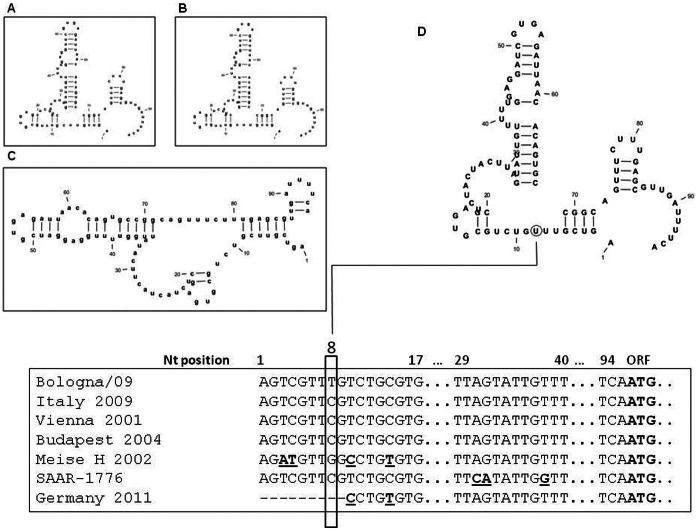
Nucleotide sequence alignment of untranslated region (UTR) of 5′ termini of different USUV strains. Proposed secondary structure of USUV strains isolated from Budapest (A), Vienna (B), South-Africa (C) and Italy from human (Bologna/09) (D), corresponding respectively to the GenBank accession numbers: **EF206350**, **AY453411**, **AY453412**, **HM569263**. The single nucleotide substitution of the human isolate contrasting with the other USUV strains of the 5′- terminus is boxed with a black line. The single nucleotide substitutions of each strain in comparison to the different USUV isolates are underlined.

Comparative analysis of the nucleotide sequences of 5′ and 3′ untranslated regions (UTR) of Bologna/09 and of the USUV sequences available from GenBank showed several nt substitutions. Within the 96-nt long 5′ UTR, one nucleotide substitution was found in the Bologna/09 strain whereas all the other sequences, from mosquito and bird derived USUV isolates did not show this nt variation, as shown in Figure 6. This single nucleotide substitution was observed at nt position 8 (Figure 6). Two distinct nucleotide substitutions were observed at nt positions 10 (C) and 14 (T) in the Meise H and Germany strains. These two nt substitutions were not detected in the other strains (Figure 6). Computer simulation of the 5′-UTR folding derived from Bologna/09 strain, produced a secondary structure model for the USUV genome. Analysis of the calculated secondary structures revealed similar folding patterns for the Bologna/09 (Figure 6D) Budapest (Figure 6/A) and Vienna (Figure 6DB) USUV strains (The Germany and Meise H isolates were presented in truncated forms). In contrast, the South-African (Figure 6C) USUV strain showed a different secondary structure. The predicted free energy of the Bologna/09, Budapest and Vienna USUV strains showed a ΔG of −21.0, −21,2 and 21,2 Kcal/mol, respectively, while the South-African strain showed a ΔG = −22,3 Kcal/ml.

**Figure 7:**
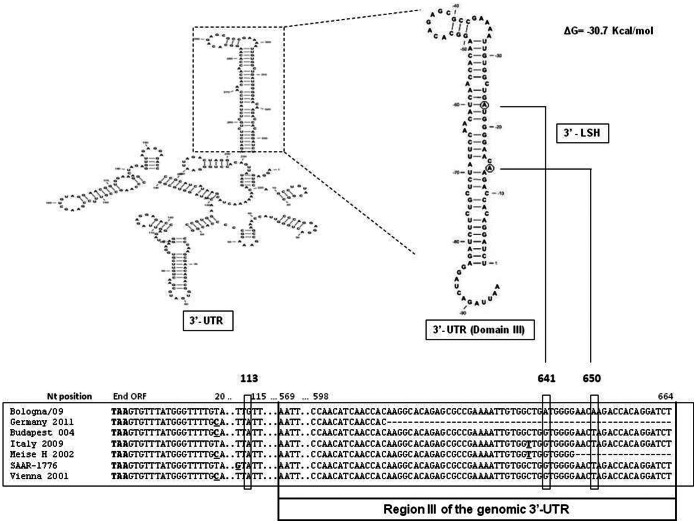
Nucleotide sequence alignment of untranslated region (UTR) of conserved 3′ termini of USUV isolates. Single nucleotide substitutions of each strain are underlined. The upper part of the figure, illustrates the predicted secondary structure of the 3′ terminal structure of Bologna/09 and the corresponding long stable hairpin (LSH) domain with the relative free energy of the structure estimated as ΔG (kcal/mol). The nucleotide substitutions from the 3′ terminus of human isolate to the different USUV strains are boxed, and their relative positions on the secondary structure are shown.

In addition, analysis of the 3′-UTR region showed three nucleotide substitutions among the different USUV isolates. In particular, specific nt substitutions for Bologna/09 were observed at positions 113, 641 and 650 (Figure 7). Among the different secondary structures present in the 3′ UTR, our attention was focused on the 3′ long stable hairpin (LSH) structure, as this has been described as a critical structure for virus viability [36].

The predicted secondary structure showed a similar folding of the 3′ LSH domain between the Bologna/09 isolate and the other USUV strains. In particular, domain III of the 3′ UTR showed a ΔG =  −38 Kcal/ml (Figure 7), while the Vienna strain (considered as the most representative isolate of the Austrian-Hungarian strains) showed a more stable predicted secondary structure (ΔG =  −39,6Kcal/ml). Based on these findings, our data suggest that the different folding free energy in the 3′ and 5′- UTRs observed between the different USUV isolates could be an important factor in the evolution of Usutu virus.

## Discussion

Following the emergence of USUV encephalitis in birds in 2001 in Europe, [4], [6], [8], [9], [11], [12] the subsequent identification of human encephalitis due to USUV in Italy, in 2009, [17]–[18] raised the possibility that this may represent the beginning of epidemic human USUV encephalitis in Europe [10]. Until very recently, epizootiological and phylogenetic evidence has supported the assumption that USUV was introduced into Austria by infected introduced mosquitoes or birds, migrating to Europe from Africa where the virus was originally assumed to circulate relatively harmlessly amongst birds, infecting humans only rarely and without evidence of encephalitis in humans [3]–[6], [10]. Recently, the evidence of USUV introduction into Europe at least since 1996, indicated that the Italian territory could be considered the epicenter of virus spread to the neighboring countries [5]. It was justifiably believed that following the introduction of USUV into Austria, it subsequently radiated from this epicenter, to neighboring countries [11]. In addition to an epidemic of chikungunya fever which was introduced into northern Italy in 2007 by an infected human returning from India [37] two other arboviruses, bluetongue virus and Schmallenberg virus, have also emerged to cause epidemics in northern Europe in recent years [38]–[39]. Consequently, in view of our interest in emerging USUV, we determined the genomic sequence of Bologna/09 virus, the first known isolate of USUV to cause human encephalitis. European isolates derived from mosquitoes and birds were compared with the Bologna/09 strain. Our data revealed a close relationship between Bologna/09 and the European avian USUV strains, with a greater divergence from the African USUV isolate. Comparative sequence analysis of the Bologna/09 human encephalitic strain identified two significant amino acid substitutions, one in the DIII-Ir domain of the E protein (S595G) and the other in the non-structural (NS) NS5 protein (D3425E). Interestingly, a similar amino acid substitution in the RdRp domain of the NS5 region, was observed in flavivirus strains that are associated with meningoencephalitis in humans (JEV, WNV, MVEV and KUNV). Importantly, studies of the closely related flavivirus WNV have shown that substitutions in virtually equivalent positions in these two genes were associated with variation in the capacity of WNV to invade the central nervous system (CNS) of laboratory mice under experimental conditions [35], [40], [41]. Domain DIII of the E protein of flaviviruses is the likely receptor-binding domain and the major determinant of virus cellular tropism, since this molecule binds to carbohydrate receptors on target cells [4244]. Specific amino acid substitutions within domain DIII have been implicated as mediators/moderators of virus infectivity, virulence, antigenicity and escape from neutralizing antibodies for WNV [40], [45]. Intriguingly, similar amino acid substitutions in the E domain were found in DENV-2 isolates in Southern-Asia, that were associated with cases of human encephalitis, whether or not they were also associated with classical clinical manifestations of dengue infection [46].

The flavivirus NS5 protein encodes the RNA dependent RNA polymerase which, together with other NS proteins and the underlying UTR skeletal backbone, is a critical component of the virus replication complex. Substitutions in the conserved domain of this polymerase, in the case of Bologna/09 (D3425E) are likely to impact, on viral replication efficiency via perturbation of the viral replication complex.

Based on our observations, we propose that the two unique substitutions identified in the Bologna/09 isolate may have played a role in the apparent altered tropism and neuroinvasive capacity of this virus for human neurological cells.

Furthermore, previous studies have drawn attention to the functional relevance of ORF secondary structures resulting from synonymous nucleotide substitutions in ssRNA viruses [23], [28], [30]. In particular, the study of Tuplin and co-workers conducted on the HCV genome, revealed that SNS in the ORF may play an important role in the folding of the RNA that impacts on essential viral processes, such as translation, replication and packaging [29]. Our analyses revealed SNS throughout the entire ORFs of the seven USUV strains and the pattern of these synonymous substitutions correlated with the geographic distribution of the USUV isolates. In particular, of the 41 SNS in the analyzed genomes of the seven USUV strains, 15 were unique for the Bologna 09 strain, another 15 were common to all Italian strains, and 4 were unique for the Austrian-Hungarian strains. Thus, the accumulation of SNS within the ORF of USUV reflects the phylogeographic separation of these viruses and appears to contribute significantly to the generation of genetic diversity and potentially, to the pathogenicity for different host species.

Extension of these observations further suggests the possibility that the SNS identified in the human Bologna/09 strain could correlate with specific changes in RNA secondary structure that result in thermo-dynamically more stable structures when compared with non-human isolates. Clearly, further biological and functional studies will be necessary to assess the significance of SNS in the determination of viral tropism, replication and pathogenicity.

The 5′ and 3′ un-translated regions (UTRs) of the USUV genome are composed of 96 and 667 bases, respectively [7]. Among the different flaviviruses, the 5′ and 3′- UTRs form terminal stem-loop sequences that play an essential role in translation and replication of the viral genome [36], [47]–[48]. Previous studies with WNV have demonstrated that single nucleotide substitutions in the 3′-UTR region can affect RNA processing and replication, as well as polyprotein synthesis and maturation during the replication cycle [49]–[50]. Among the different functional regions within the 3′- UTR, region III has been the most studied [25], [36] because it forms a long stable hairpin (LSH) that is conserved in all the flaviviruses studied thus far [36], [48]. It is important to note that in our study, specific nucleotide substitutions were identified in the LSH region of the 3′ - UTR of the strain Bologna/09 and that these substitutions were not identified amongst the other USUV strains for which complete genome sequence is presently available. The two nucleotide positions where the sequence was detected in the Bologna/09 strain genome (nt 641 and 650) were located in the 3′ region of the 3′ terminal structure. It needs to be emphasized that, with the exception of the Bologna/09 strain, this is a completely conserved sequence within the LSH in the different USUV genomes. Previous studies showed that the specific binding site for eukaryotic translation elongation factor (eEF1A) is located in the LSH domain within the 3′ - UTR and that nucleotide substitutions in this region decreased eEF1A binding in *in vitro* assays [25], [50]. The eEF1A delivers the aminoacylated tRNAs and the GTP to the A site on the ribosome during protein synthesis [25], [50], thus mediating an important function of the viral replication process.

Blackwell and co-workers demonstrated that a single nucleotide substitution within this region was responsible for molecular re-orientation leading to increased interaction between EF1a and the 3′ SLH viral RNA in WNV and increased WNV replication [24], [49], [51].

Analysis of the RNA folding free energy both on the 5′ and 3′-UTRs observed from different strains suggests divergent evolution of USUV. Furthermore, these data could indicate that the RNA folding free energy of the UTR regions could have arisen following host adaptation of USUV from birds to humans possibly under the selective influence of the different temperatures in humans and birds.

Perhaps the key message of this study is that the genomic sequence of the human Bologna/09 USUV isolate exhibits several specific differences when compared with the genomes of USUV isolates from mosquitoes or birds. We recognize that our results are based on the identification and isolation of a single human case of clinically neuroinvasive disease. However, in summary, we have identified two amino acid substitutions, one in the DIII domain of the E protein and one in the RdRp conserved domain of the NS5 protein, together with unique synonymous substitutions throughout the genome.

In conclusion, whilst more evidence will be required, the concept that a human neurovirulent strain of USUV with unique genetic characteristics has been identified in northern Italy requires serious further consideration in view of the potential public health implications.

## Supporting Information

Table S1 **Comparison of the nucleotide and corresponding amino-acid substitutions between Bologna/09 (human isolate) and African (SAAR-1776 strain) and European (Vienna 2001, Meise H 2002, Budapest 2005, Italia 2009 and Germany 2011) USUV isolates.**
(DOC)
