# Supplementary material for: Comparative Genomic and Phylogenetic Analysis of the First Usutu Virus Isolate from a Human Patient Presenting with Neurological Symptoms
Source: PLoS One. 2013 May 31;8(5):e64761. doi: 10.1371/journal.pone.0064761 (PMC3669420; doi:10.1371/journal.pone.0064761)
Supplement: Table S1 — Comparison of the nucleotide and corresponding amino-acid substitutions between Bologna/09 (human isolate) and African (SAAR-1776 strain) and European (Vienna 2001, Meise H 2002, Budapest 2005, Italia 2009 and Germany 2011) USUV isolates. (DOC) [file pone.0064761.s001.doc]

**Table S1.** Comparison of the nucleotide and corresponding amino-acid substitutions between Bologna/09 (human isolate) and African (SAAR-1776 strain) and European (Vienna 2001, Meise H 2002, Budapest 2005, Italia 2009 and Germany 2011) USUV isolates.

|  | **Codon modification**  **[USUV Bologna/09]_aa_ [USUV Reference Strain]_aa_** | | | | | | | | | | |
| --- | --- | --- | --- | --- | --- | --- | --- | --- | --- | --- | --- |
|  | **Structural Protein** | | | **NonStructural Protein** | | | | | | | |
|  | **C** | **M** | **E** | **NS1** | **NS2a** | **NS2b** | **NS3** | **NS4a** | **2K** | **NS4b** | **NS5** |
| **Reference**  **Strains** |  |  |  |  |  |  |  |  |  |  |  |
|  |  |  |  |  |  |  |  |  |  |  |  |
| **SAAR-1776**  **(AY453412)** | [GTT]**_Val_**  [GCC]**_Ala120_** |  | [ATG]**_Met_** [GTG]**_Val419_**  [GGC]**_Gly_** [AGC]**_Ser_** 569  **^a^[AGC]_Ser_ [GGC]_Gly595_**  [GGC]**_Gly_** [AGC]**_Ser613_**  [ACA]**_Thr_** [CCA]**_Pro716_**  [AAC]**_Asn_** [AGC]**_Ser790_** | **^b^[GGA]_Gly_ [GAG]_Glu830_**  [AGG]**_Arg_** [AAG]**_Lys1117_** | [AAC]**_Asn_**  [GAC]**_Asp1267_**  [TTT]**_Phe_** [CTC]**_Leu1268_**  [ACC]**_Thr_**  [ATT]**_Ile1324_** |  | **^b^[GTA]_Val_ [ATA]_Ile1602_**  [ATC]**_Ile_** [GTC]**_Val1618_**  [GCT]**_Leu_** [GAT]**_Met1695_**  [AGA]**_Arg_** [ATA]**_Ile2030_**  [GAG]**_Glu_** [CAG]**_Gln2032_**  [GCT]**_Ala_** [TCC]**_Ser2103_** | [CTT]**_Leu_**  [TTT]**_Phe2166_** |  | [ATA]**_Ile_** [ATG]**_Met2287_**  [AGC]**_Ser_** [GGC]**_Gly2290_**  **^b^[AGC]_Ser_ [AAC]_Asn2304_** | [AAG]**_Lys_** [AGG]**_Arg2550_**  **^b^** [ATA]**_Ile_** [ATG]**_Met2645_**  [ACC]**_Thr_** [GCC]**_Ala2803_**  [AGC]**_Ser_** [GGC]**_Gly2849_**  **^a^[GAT]_Asp_ [GAA]_Glu3425_**  [AAT]**_Asn_** [GAT]**_Asp3427_** |
|  |  |  |  |  |  |  |  |  |  |  |  |
|  |  |  |  |  |  |  |  |  |  |  |  |
| **Vienna 2001**  **(AY453411)** |  |  | **^a^[AGC]_Ser_ [GGC]_Gly595_** | **^b^[GGA]_Gly_ [GAA]_Glu830_**  [GTA]**_Val_** [GCA]**_Ala939_** | [TAT]**_Tyr_** [CAT]**_Hys1146_**  [ACC]**_Thr_** [ATC]**_Ile1324_** |  | **^b^[GTA]_Val_ [ATA]_Ile1602_**  [GTT]**_Val_** [GCT]**_Ala1779_** |  |  | **^b^[AGC]_Ser_ [AAC]_Asn2304_**  [TTG]**_Leu_** [TTT]**_Phe2367_** | **^b^** [ATA]**_Ile_** [ATG]**_Met2645_**  **^a^[GAT]_Asp_ [GAA]_Glu3425_** |
|  |  |  |  |  |  |  |  |  |  |  |  |
|  |  |  |  |  |  |  |  |  |  |  |  |
| **Meise H 2002**  **(JQ219843)** |  |  | **^a^[AGC]_Ser_ [GGC]_Gly595_** | [GTC]**_Val_** [ATC]**_Ile822_**  **^b^[GGA]_Gly_ [GAA]_Glu830_**  [GTA]**_Val_** [GCA]**_Ala939_** | [TAT]**_Tyr_** [CAT]**_Hys1146_**  [ACC]**_Thr_** [ATC]**_Ile1324_** |  | **^b^[GTA]_Val_ [ATA]_Ile1602_** |  |  | **^b^[AGC]_Ser_ [AAC]_Asn2304_** | **^b^**[ATA]**_Ile_** [ATG]**_Met2645_**  **^a^[GAT]_Asp_ [GAA]_Glu3425_** |
|  |  |  |  |  |  |  |  |  |  |  |  |
|  |  |  |  |  |  |  |  |  |  |  |  |
| **Budapest 2005**  **(EF206350)** |  | [ACT]**_Thr_**  [ATT]**_Ile176_** | **^a^[AGC]_Ser_ [GGC]_Gly595_** | **^b^[GGA]_Gly_ [GAA]_Glu830_**  [GTA]**_Val_** [GCA]**_Ala939_** | [TAT]**_Tyr_** [CAT]**_Hys1146_** |  | **^b^[GTA]_Val_ [ATA]_Ile1602_** |  |  | **^b^[AGC] _Ser_ [AAC] _Asn2304_** | **^b^**[ATA]**_Ile_** [ATG]**_Met2645_**  **^a^[GAT]_Asp_ [GAA]_Glu3425_** |
|  |  |  |  |  |  |  |  |  |  |  |  |
|  |  |  |  |  |  |  |  |  |  |  |  |
| **Italia 2009**  **(JF266698)** |  | [AAA]**_Lys_** [AGA]**_Arg181_** | **^a^[AGC]_Ser_ [GGC]_Gly595_** |  |  |  |  |  |  |  | **^a^[GAT]_Asp_ [GAA]_Glu3425_** |
|  |  |  |  |  |  |  |  |  |  |  |  |
|  |  |  |  |  |  |  |  |  |  |  |  |
| **Germany 2011**  **(HE599647)** | [AAC]**_Asn_** [AGC]**_Ser11_**  [AAA]**_Lys_** [AGA]**_Arg85_** |  | **^a^[AGC]_Ser_ [GGC]_Gly595_** | **^b^[GGA]_Gly_ [GAA]_Glu830_** | [GTC]**_Val_** [ATC]**_Ile1219_**  [GCA]**_Ala_** [GTA]**_Val1237_**  [ACC]**_Thr_** [ATC]**_Ile1324_** |  | **[CTT]_Leu_ [TTT]_Phe1549_**  **^b^**[GTA]**_Val_** [ATA]**_Ile1602_** |  |  | **^b^[AGC]_Ser_ [AAC]_Asn2304_**  [AAG]**_Lys_** [AGA]**_Arg2445_** | **^b^** [ATA]**_Ile_** [ATG]**_Met2645_**  **^a^[GAT]_Asp_ [GAA]_Glu3425_** |
|  |  |  |  |  |  |  |  |  |  |  |  |

**^a^** aminoacids substitution of USUV Bologna/09 (human isolates) in comparison to not human USUV reference strains

**^b^** aminoacids subtitution of USUV italian isolates in comparison to the European and African USUV strains
